# A Very Rare Complication of Hepatitis A Infection: Acute Myocarditis—A Case Report with Literature Review

**DOI:** 10.1155/2018/3625139

**Published:** 2018-09-13

**Authors:** Olivia Allen, Ahmed Edhi, Adam Hafeez, Alexandra Halalau

**Affiliations:** ^1^Internal Medicine Department, Beaumont Hospital, Royal Oak, MI, USA; ^2^Oakland University William Beaumont School of Medicine, Rochester, MI, USA

## Abstract

Hepatitis A is a common viral infection with a benign course but in rare cases can progress to acute liver failure. It usually presents with abdominal pain, nausea, vomiting, diarrhea, jaundice, anorexia, or asymptomatically, but it can also present atypically with relapsing hepatitis and prolonged cholestasis. In addition, extrahepatic manifestations have been reported, including urticarial and maculopapular rash, acute kidney injury, autoimmune hemolytic anemia, aplastic anemia, acute pancreatitis, mononeuritis, reactive arthritis, glomerulonephritis, cryoglobulinemia, Guillain–Barre syndrome, and pleural or pericardial effusion. A rare manifestation of hepatitis A is acute myocarditis. We report a case of a young woman who presented with “flu-like symptoms” and was found to have severe elevation of liver enzymes due to acute hepatitis A infection. On her 3rd day of admission, the patient developed chest pain and nonspecific electrocardiographic changes. Her troponins rose to 16.4  ng/mL, and a transthoracic echocardiogram revealed global hypokinesis and a depressed ejection fraction at 30%. A CT angiography showed no evidence of significant coronary artery disease. The patient was managed supportively, and symptoms and laboratory findings slowly improved over the next 7 days. Her chest pain resolved and a follow-up echocardiogram showed improved ejection fraction to 45%.

## 1. Introduction

Hepatitis A is a common viral illness caused by the hepatitis A virus (HAV). Although avoidable by immunization, it is the most common infection leading to liver disease worldwide [1]. HAV is fecal-orally transmitted and is endemic in developing countries [2]. The incidence of hepatitis A has increased in the United States due to outbreaks. Since March 2017, 694 individuals were affected in California, with 454 hospitalizations and 21 deaths, and it is considered to be the largest outbreak in the United States in the last two decades. Since August 2016, there have been 790 cases in Michigan, with 615 hospitalizations and 25 deaths. Other states affected include Kentucky and Utah [3].

Clinical features are more common with increasing age [2]. Symptom onset follows a 2- to 7-week incubation period. Due to viral shedding, patients are infectious from the beginning of the incubation period until one week after resolution of jaundice. Common features include fever, fatigue, abdominal pain, nausea, emesis, and jaundice. The illness is usually self-limited and does not lead to chronic disease. Complications include cholestatic hepatitis, relapsing hepatitis, and autoimmune hepatitis. Rarely, hepatitis A can progress to acute liver failure. Extrahepatic manifestations may include urticarial and maculopapular rash, acute kidney injury, autoimmune hemolytic anemia, aplastic anemia, acute pancreatitis, mononeuritis, reactive arthritis, Guillain–Barre syndrome and pleural or pericardial effusion, glomerulonephritis, polyarteritis nodosa, cryoglobulinemia, and thrombocytopenia [4, 5]. Myocarditis is a very rare extrahepatic complication with only three reported cases [6–8].

Myocarditis is an inflammatory disease of the myocardium [9]. Myocarditis can lead to acute heart failure, chronic dilated cardiomyopathy, and sudden cardiac death. Viral myocarditis is the most common cause of acquired myocarditis with parvovirus B19 and adenovirus being the most commonly identified [10]. Enteroviruses, variola, influenza, and herpesviruses are well recognized but not common. Although relatively rare, human immunodeficiency virus and hepatitis C and B have also been implicated [11]. Myocardial injury is due to the host immune response against the virus and possibly related to viral proliferation in cells. Damage to cardiac myocytes can lead to impaired contractility, ventricular wall stiffening, or conduction abnormalities [12]. Myocarditis can present asymptomatically or with chest pain, dyspnea, and palpitations, all of which mimic more common disorders. ECG findings may include ST-T wave changes and conduction delays [12]. Troponin levels can be elevated [13].

## 2. Case Report

A 30-year-old Caucasian woman, without significant past medical history, presented to our hospital with “flu-like symptoms.” She admitted to four days of fatigue, myalgias, nausea, nonbilious emesis, and nonbloody diarrhea. She complained of abdominal pain, anorexia, and dark urine. She took no medications and family history was noncontributory. She denied tobacco and illicit drug use and admitted to rare alcohol use. She worked as a substitute teacher for elementary-aged children. She denied recent travel and lived in the United States for the duration of her life. She admitted to frequenting multiple restaurants. She denied sick contacts. On arrival to the emergency department, her blood pressure was 120/64 mmHg, heart rate was 49 beats per minute (bpm), respiratory rate was 20 breaths per minute, and temperature was 36.4°C. Physical examination was significant for mild scleral icterus and right upper quadrant abdominal tenderness, without peritoneal signs or fluid wave that would indicate ascites. She had intact mentation, no asterixis, and no stigmata of liver disease including no palmar erythema and no telangiectasias. Skin examination revealed no rash.

Lab work revealed severe elevations in liver transaminases, aspartate aminotransferase (AST) was 6,769 U/L, alanine aminotransferase (ALT) was 8,479 U/L, INR was elevated at 2.0, and acute viral hepatitis panel was positive for hepatitis A IgM only. EBV IgG and IgM were positive, but heterophile assay and EBV PCR was negative (Table 1). Limited abdominal ultrasound revealed diffuse gallbladder wall thickening and edema with trace-free fluid in the right upper quadrant and no evidence of stones, sludge, or sonographic Murphy's sign, with findings consistent with hepatocellular disease. Hepatic echotexture was homogenous without evidence of focal hepatic lesions. There was mild intrahepatic biliary ductal dilatation with common bile duct measuring 2 mm. A mildly enlarged peripancreatic lymph node was also seen. No pancreatic ductal dilatation was present. Supportive care was initiated with intravenous fluids and close monitoring of her laboratory values. A urinalysis revealed orange, turbid urine positive for bilirubin, 15 mg/dL ketones, 30 mg/dL of protein, 3+ blood with 11–24 RBCs, >50 WBCs, 4+ bacteria, 2+ leukocyte esterase, no nitrites, no casts, and >50 squamous cells. She was started on IV ceftriaxone for presumed urinary tract infection.

**Figure 1:**
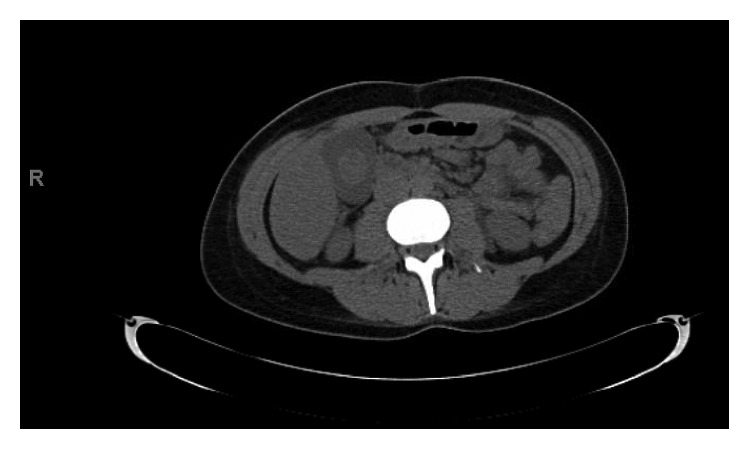
CT abdomen and pelvis without contrast demonstrating mild perihepatic ascites, diffuse low-attenuation of the liver likely due to active hepatocellular disease. Diffusely thickened gallbladder wall as demonstrated on abdominal ultrasound. Additional CT findings include a moderate amount of free fluid in the pelvis and a small right-sided pleural effusion. No obstructing renal calculus identified.

**Figure 2:**
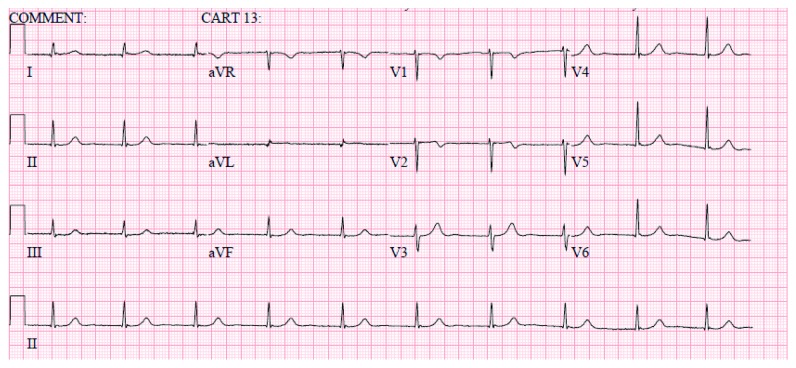
ECG obtained on day 3 exhibiting a heart rate of 60 bpm, a junctional rhythm, normal axis, and T wave inversions in V1 and V2.

**Figure 3:**
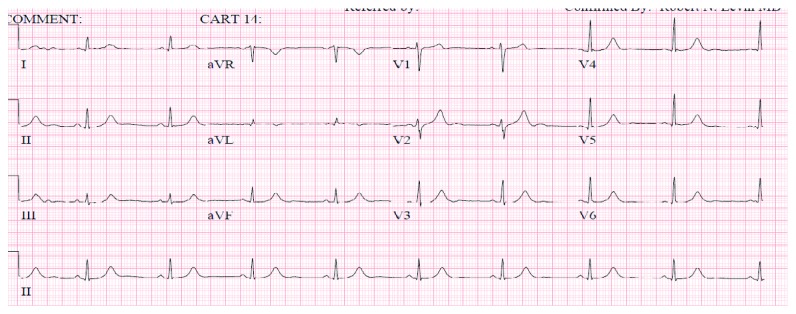
ECG from the same patient in normal sinus rhythm. T wave inversions not evident in V1 and V2.

On day 2, the patient developed severe left-sided flank pain. CT abdomen pelvis revealed no obstructing renal calculus but redemonstrated diffuse gallbladder wall thickening felt to be secondary to hepatitis. There was diffuse low-attenuation of the liver and perihepatic ascites, a moderate amount of free fluid in the pelvis, and a small right-sided pleural effusion (Figure 1). Her pain worsened, and she complained of diaphoresis and lightheadedness. She was found to be bradycardic with heart rates as low as 30 s. Blood pressure was within normal limits. ECG revealed sinus bradycardia only. Troponin was negative. On day 3, she developed chest pain described as a burning sensation in the midepigastric region. Pain was relieved with an antacid, and a proton pump inhibitor was initiated. ECG revealed a rate of 60 bpm, a junctional rhythm, normal axis, and new T wave inversions in V1 and V2 (Figure 2). An ECG without these abnormalities obtained prior is also shown (Figure 3). Troponin was elevated at 6.21, trended up to 10.60, and the patient was transferred to the cardiac intensive care unit. Hepatic panel revealed improvement in transaminases (Table 2).

**Figure 4:**
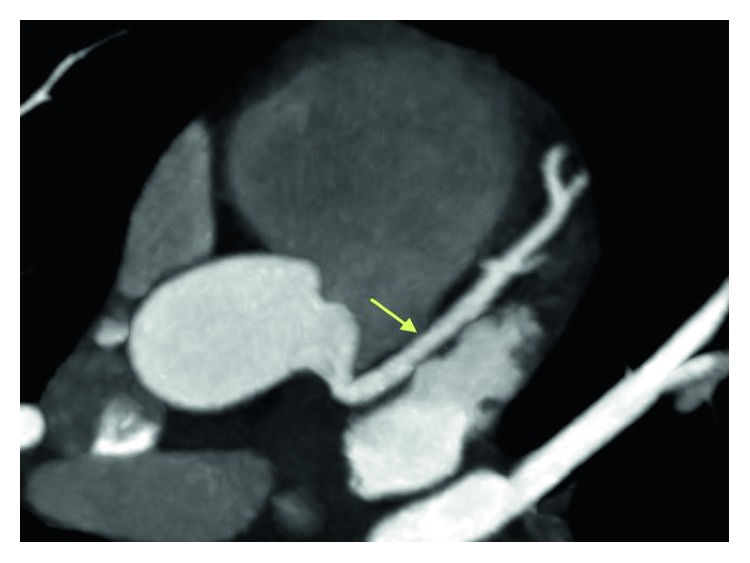
Coronary CT angiogram image revealing normally positioned left anterior descending artery (LAD) without any evidence of coronary artery disease.

**Figure 5:**
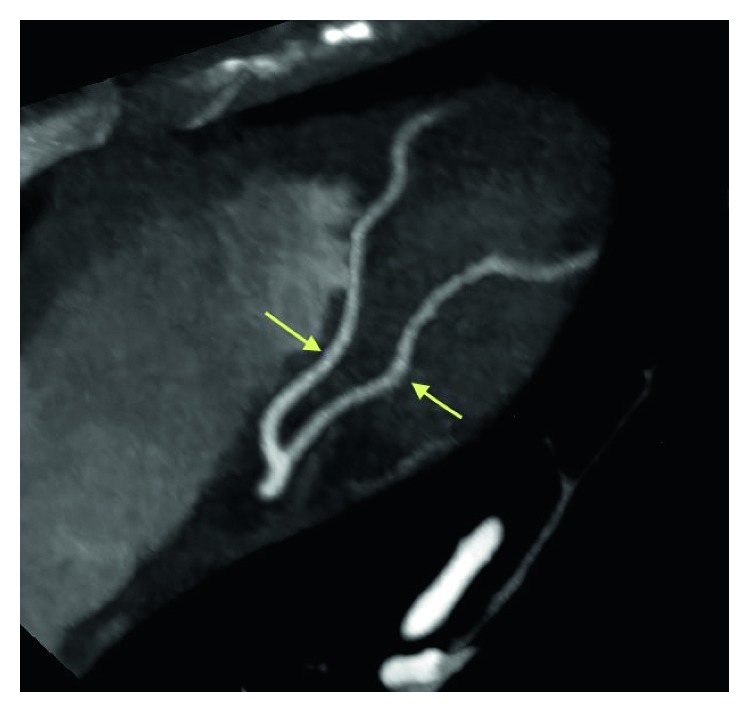
Coronary CT angiogram image revealing the distal segments and diagonal branches of LAD are normal without any evidence of coronary artery disease.

**Figure 6:**
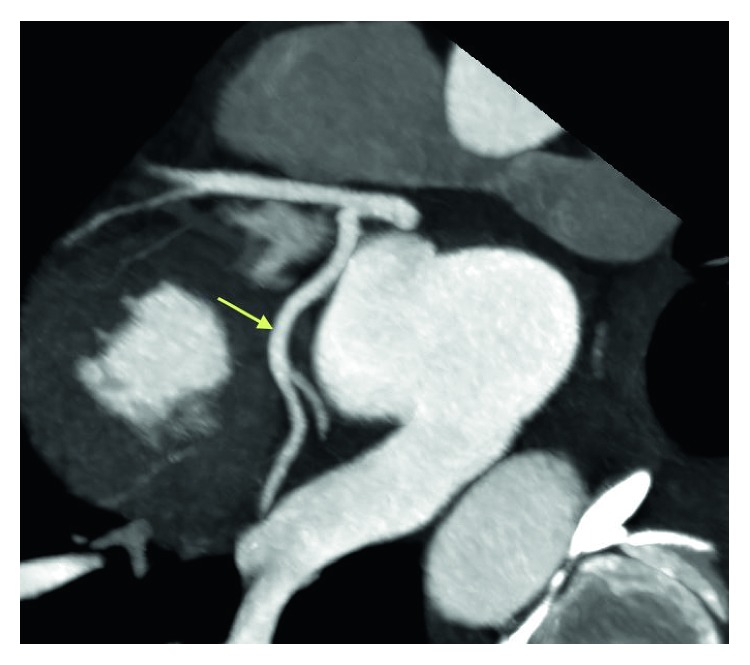
Coronary CT angiogram image revealing the circumflex is nondominant. The proximal segment and obtuse marginal branches are normal.

**Figure 7:**
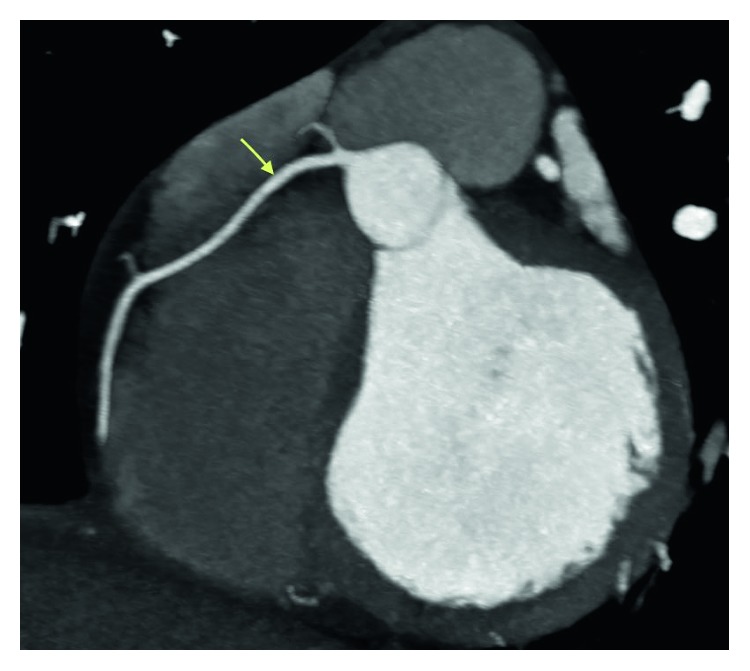
Coronary CT angiogram image revealing the right coronary artery arises normally from the right coronary cusp and is dominant without any evidence of coronary artery disease.

She received aspirin and started on IV heparin. Repeat troponin was 16.4. A transthoracic echocardiogram revealed diffuse global hypokinesis of the left ventricle. The estimated ejection fraction was 30% without evidence of pericardial effusion. A CT angiography of her coronaries revealed normal coronary arteries, aorta, and pericardium (Figures 4–7). IV heparin was discontinued. A presumed diagnosis of myocarditis was made.

**Figure 8:**
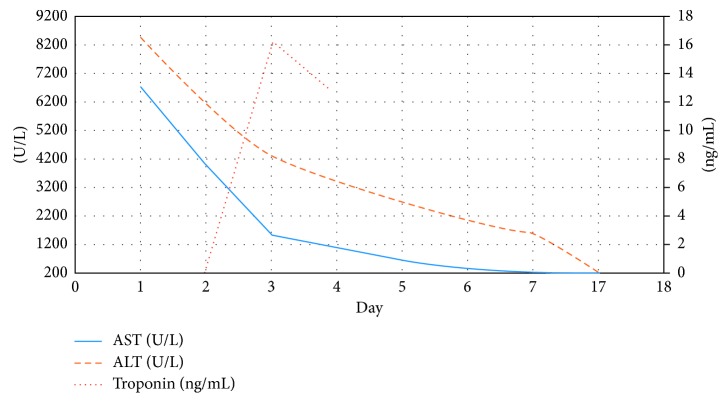
Trend of liver function tests and troponin obtained during hospital course. Day 1 is day of admission. Day 17 is outpatient follow-up.

The patient continued to feel better, troponins began to trend down (Table 3), and hepatic indices improved (Figure 8). A follow-up echocardiogram revealed an improved ejection fraction of 45%. Our patient was discharged home with plan for follow-up to continue monitoring labs. Two months after discharge, a repeat echocardiogram was obtained revealing an ejection fraction of 56%, normal left ventricular size and systolic function, and no wall motion abnormalities.

## 3. Discussion

Most hepatitis A–infected adults manifest with severe hepatitis with markedly elevated liver enzymes. Elevations in transaminases are >1000 s IU/dL, with ALT usually greater than AST. Transaminases precede elevations in bilirubin, with bilirubin usually 10 mg/dL or less [14]. Progression to fulminant hepatitis is rare, <1% [4], and the illness is usually self-limited. There is no guideline or recommendation on the treatment of hepatitis A. General supportive measures and avoidance of liver toxins are recommended. If acute liver failure is present, then liver transplantation needs to be considered [4].

There are rare extrahepatic manifestations of acute hepatitis A, from which myocarditis has been reported extremely rare. To our knowledge, there are 3 case reports of myocarditis associated with hepatitis A (Table 4), but none reported in the United States, where hepatitis A infection is relatively less common. Two possible additional cases were unable to be translated to English and so will not be discussed.

We have presented a case of severe hepatitis A leading to acute myocarditis. Like previous case reports, a presumptive diagnosis of myocarditis secondary to hepatitis A was made, despite endomyocardial biopsy (EMB) being the gold standard for definitive diagnosis. The World Health Organization (WHO) and International Society and Federation of Cardiology (ISFC) define myocarditis as inflammation of the myocardium that meets histological, immunological, and immunohistochemical criteria [15]. However, EMB is not always indicated especially if it would not change management. Our patient did not meet the American Heart Association/American College of Cardiology Foundation/European Society of Cardiology (AHA/ACC/ESC) guideline for EMB which includes patients with new onset heart failure with hemodynamic compromise or those with new onset heart failure who also have a dilated left ventricle or new ventricular arrhythmias, second- or third-degree heart block, or failed to respond to usual care in 1–2 weeks [16]. Given that our patient had an acute viral illness, nonspecific ECG changes, troponin elevations with normal CT angiography, and an echocardiogram with left ventricular systolic dysfunction, there is enough clinical evidence to suspect myocarditis in this patient. EBV IgM and IgG were positive; however, EBV nuclear antigen (EBNA) was also positive. EBNA develops 3–6 weeks after symptoms onset; therefore, positivity excludes acute primary EBV. Positivity can be seen in reactivation of EBV, but EBV PCR testing was done and was negative. Additionally, our patient was without fever, pharyngitis, and lymphadenopathy, and the degree of hepatitis was more consistent with acute hepatitis A. Given the pattern of EBV positivity, negative EBV PCR, and our patient's presentation, the presence of these antibodies most likely represents seropositivity due to acute inflammation and infection in the setting of severe acute hepatitis A and not reactivation of EBV [17].

Despite improving liver indices, our patient developed myocarditis. Therefore, it may be worthwhile to monitor patients for cardiac abnormalities despite signs of improving transaminases. In the case reported by Park et al. [6] ECG changes preceded troponin elevations. They suggest obtaining echocardiography in the setting of ECG changes even if troponin is not elevated. This differs from our case in that troponin elevation coincided with our patient's symptoms and nonspecific ECG changes, indicating a variable timeline to injury. The patient reported by Park et al. [6] had milder elevations in transaminases, with milder troponin elevation and a decreased ejection fraction to only 52% [6]. In the case reported by Jagtap et al. [7] their patient had higher elevations in transaminases like our patient and similar elevations in bilirubin and INR, with a larger depression in ejection fraction to 35%. Precise level of troponin elevation was not provided [7]. In the third case, reported by Botero et al. [8], their patient had milder elevations of transaminases, but had a more severe coagulopathy with an INR >10, higher elevations of bilirubin, and required liver transplantation. Their patient's cardiomyopathy was milder with an ejection fraction of 59%. No troponin level was mentioned [8]. Based on these comparisons, higher elevations in transaminases may be associated with more severe cardiomyopathy. However, given the rarity of this manifestation, true associations are yet to be elucidated.

The mechanism of myocyte injury with hepatitis A is not yet known. Currently, viral myocarditis is thought to be due to the immune response to myocyte infiltration of the virus, of which certain viruses seem to have a cardiac predilection. Presentation varies from asymptomatic to fulminant heart failure. Myocarditis may present like a myocardial infarction with angina, dyspnea, arrhythmias, ECG changes, and troponin elevations [9]. Most patients have nonspecific symptoms related to the viral illness, but there is risk for progression to dilated cardiomyopathy [18]. The natural history of viral myocarditis is not well defined and progression to dilated cardiomyopathy is highly variable with up to 30% of biopsy-proven cases reported [9]. Etiology of myocarditis, clinical presentation, and disease stage are factors that have been shown to predict prognosis [9, 18]. For example, in patients with enterovirus or parvovirus B19 myocarditis, persistence of the viruses in the myocardium and endothelial cells, respectively, were associated with ventricular dysfunction and poorer outcomes compared to those who cleared the virus [9]. Most patients with Coxsackie B virus present subclinically and usually have a benign course [19]. Patients with giant cell or eosinophilic myocarditis have a poorer prognosis with reports showing survival of less than 20% at 4 years. Given these findings, awareness of hepatitis A as a cause of myocarditis may be important for prognosis. More research into the natural history of hepatitis A myocarditis would need to be done to predict prognosis.

In conclusion, we have demonstrated a rare case of myocarditis associated with acute hepatitis A infection. The purpose of this case report is to increase awareness of this disease entity so it can be recognized and considered in clinical practice.

## Figures and Tables

**Table 1:** Key laboratory values on admission.

Laboratory	Value	Normal
Hemoglobin/hematocrit	15.8 g/dL/45.5%	12.1–15.0 g/dL
Platelets	148 g/L	150–400 bil/L
AST (U/L)	6,769	10–37
ALT (U/L)	8479	8–37
ALP (U/L)	149	30–110
Albumin (g/dL)	3.7	3.5–5.1
Total bilirubin (mg/dL)	7.1	0.3–1.2
Direct bilirubin (mg/dL)	4.2	0.0–0.3
INR	2.0	1.0
Calcium (mg/dL)	9.1	8.4–10.4
Fibrinogen (mg/dL)	143	175–375
Acetaminophen level (mcg/mL)	<3.0	0.0–30.0
Acute hepatitis panel	HAV IgM positive	Negative
Partial thromboplastin time (seconds)	40.4	23.0–30.0
Lipase (U/L)	71	7–60
Blood urea nitrogen (mg/dL)	19	8–22
Creatinine (mg/dL)	0.79	0.60–1.40
Glomerular filtration rate (GFR) (mL/min/1.73 m^2^)	100	>59
Factor V activity	67	60–120%
Lactic acid	5.8, 2.4 mg/dL	0.5–2.2 mmol/L
Heterophile antibody	Negative	Negative
EBV IgM (U/mL)	56.1	0–35.9
EBV nuclear Ag (U/mL)	499.0	0–17.9
EBV IgG (U/mL)	>750	0–17.9
EBV early Ag (U/mL)	44.8	0–8.9
EBV by PCR	Not detected	Not detected

AST =  aspartate aminotransferase; ALT =  alanine aminotransferase; ALP = alkaline phosphatase.

**Table 2:** Trend of liver function tests.

Laboratory	Day 1	Day 2	Day 3	Day 4	Day 5	Day 6	Day 7	Day 17
Aspartate aminotransferase (U/L)	6,769	4,058	1,603	1,117	683	421	276	206
Alanine aminotransferase (U/L)	8479	6,168	4,327	3,482	2,729	2,166	1,658	338
Alkaline phosphatase (U/L)	149	129	103	109	123	141	147	186
Albumin (g/dL)	3.7	3.1	2.6	2.5	2.6	2.8	2.7	4.3
Total bilirubin (mg/dL)	7.1	7.4	5.2	7.0	9.3	10.8	9.7	4.9
Direct bilirubin (mg/dL)	4.2	4.5	4.1	5.4	7.2	8.4	7.6	—
INR	2.0	2.1	1.7	1.5	1.3	—	—	—

**Table 3:** Troponin trend.

Day 2 (ng/mL)	Day 3 (ng/mL)			Day 4 (ng/mL)		Normal (ng/mL)
<0.03	6.21	10.60	16.40	14.70	12.50	0.00–0.05

**Table 4:** Other reported cases of myocarditis in hepatitis A.

Author, year	Botero et al. [8], 2017	Park et al. [6], 2011	Jagtap et al. [7], 2008
Age, gender	10, M	33, F	19, M
Peak key liver laboratory values	AST = 378 U/L ALT = 280 U/L Bilirubin = 26.9 mg/dL	AST = 172 U/L ALT = 214 U/L Bilirubin = 3.7 mg/dL	ALT = 7080 U/L Bilirubin = 9.2 mg/dL
Peak troponin	Not reported	2.17 ng/mL	Reported as “positive”
Cardiac symptoms	None reported	None reported	None reported
ECG changes	Bradycardia, low voltage, and 3rd-degree AV block	Sinus tachycardia and mild T wave inversions in II, III, aVF, and V2–V6	Sinus tachycardia
Ejection fraction	59%	52% with LV global hypokinesis	35% with septum and posterior wall severely hypokinetic
Time to resolution	5 days after liver transplant	9 days after admission	All investigations normal at one-month follow-up

AST =  aspartate aminotransferase; ALT =  alanine aminotransferase; ALP = alkaline phosphatase; F = female; M = male.
